# Supplementary material for: Global geo-hazard risk assessment of long-span bridges enhanced with InSAR availability
Source: Nat Commun. 2025 Oct 13;16:9048. doi: 10.1038/s41467-025-64260-x (PMC12518672; doi:10.1038/s41467-025-64260-x)
Supplement: Supplementary file 1 — Supplementary Information [file 41467_2025_64260_MOESM1_ESM.pdf]

Supplementary Information

Global Geo-hazard Risk Assessment of Long-Span Bridges Enhanced with InSAR Availability

Dominika Malinowska[1,2,*], **Pietro Milillo**[3,4,5], **Cormac Reale**[2], **Chris Blenkinsopp**[2], **and Giorgia Giardina**[1]

[1]Delft University of Technology, Department of Geoscience & Engineering, Delft, 2628 CD, The Netherlands
[2]University of Bath, Department of Architecture and Civil Engineering, Bath, BA2 7AY, UK
[3]University of Houston, Cullen College of Engineering, Department of Civil and Environmental Engineering, Houston, TX 77204-4007, USA
[4]University of Houston, Cullen College of Engineering, Department of Earth and Atmospheric Science, Houston, TX 77204-4007, USA
[5]German Aerospace Center, Microwaves and Radar Institute, Weßling, 82234, Germany
[*]D.U.Malinowska@tudelft.nl

Results

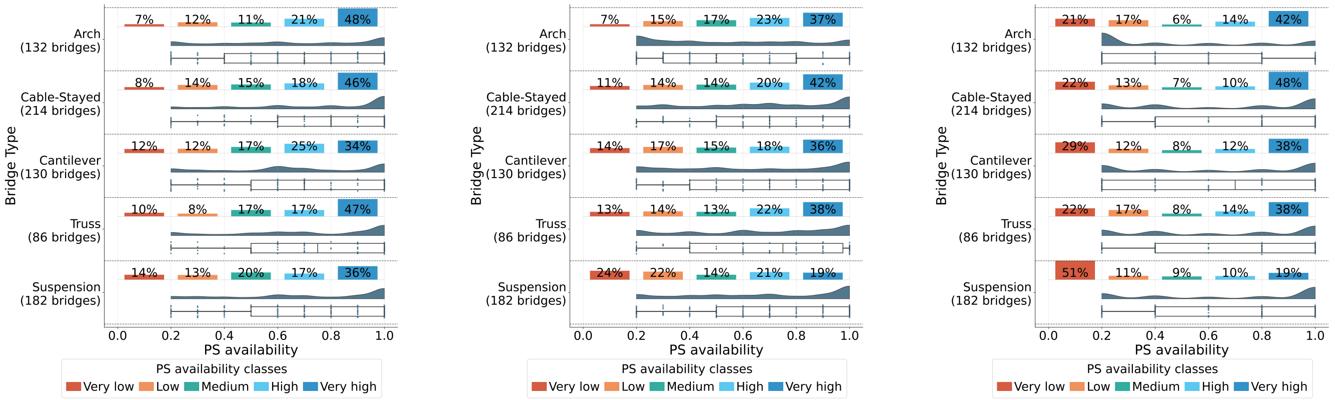

(a) Edge spans. **(b)** Intermediate spans. **(c)** Central span.

Supplementary Figure 1. Distribution of PS availability depending on bridge type, showing differences in distribution between spans. As there are two Edge and Intermediate spans, the mean was taken for each bridge.

Methods

Long-span bridges database

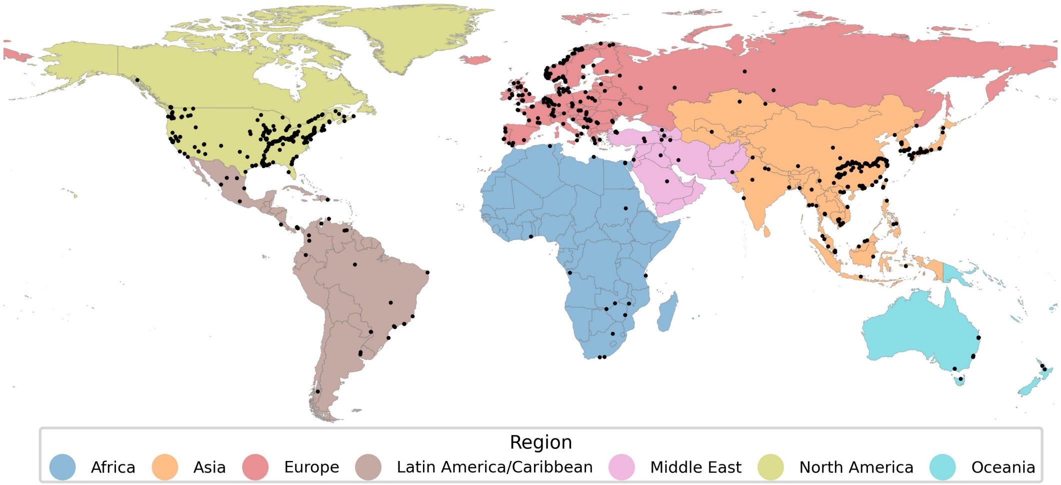

Supplementary Figure 2. Map showing the location of all bridges from the database employed in this study[1]. The region division follows the one proposed in the original database.

Hazards

Supplementary Table 1. Translation of subsidence hazard values.

**Scale in[2]**	**Ordinal Value**	**Normalised Values**
No data	15	0
Very Low	1	0.1667
Low	2	0.334
Medium Low	3	0.5
Medium High	4	0.6667
High	5	0.8333
Very High	6	1

Supplementary Table 2. Translation of landslide hazard values.

**Scale in[3]**	**Ordinal Value**	**Normalised Values**
No data	255	0
Very Low	1	0.25
Low	2	0.5
Medium	3	0.75
High	4	1

Exposure

Supplementary Table 3. Exposure classes based on the type of transportation network carried by the bridge.

Exposure class	Ordinal value (Normalised)	Bridge functionality
High	5 (1)	Highway-railway dual function bridge
Medium-high	4 (0.8)	Rail or motorway road
Medium	3 (0.6)	Trunk or primary roads
Medium-low	2 (0.4)	Secondary road
Low	1 (0.2)	Footway or other

Structural Vulnerability

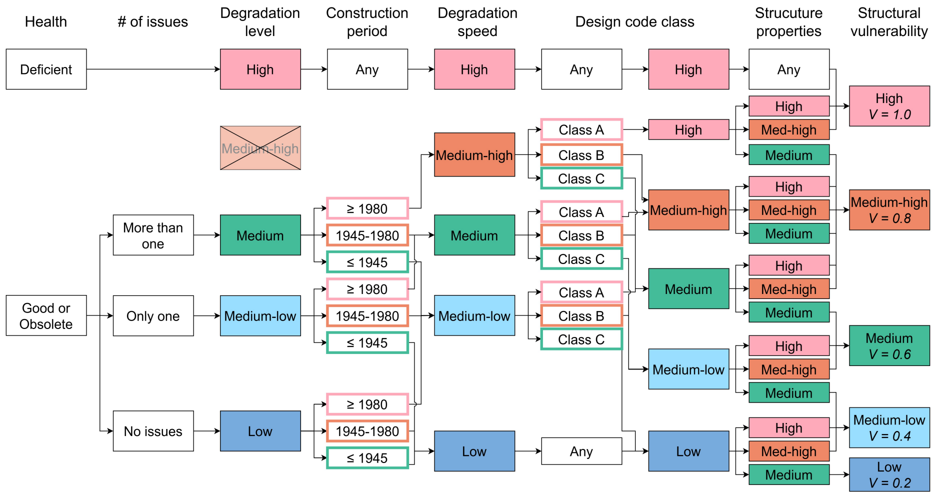

Supplementary Figure 3. Logical process for assigning structural vulnerability classes to each bridge, simplified from the Italian guidelines to include only relevant categories. Note that younger bridges are assigned higher degradation speeds following the guidelines' assumption that bridges experiencing rapid deterioration relative to their age are relatively more vulnerable.

Supplementary Table 4. Assumptions used to assign structural vulnerability classes from the Italian Guidelines to long-span bridge types not explicitly covered in the original guidelines.

Bridge type	Assumptions and rationale	Material	Assigned class
Arch	The classification "Arco sottile" (thin arch) was applied as worst-case scenario. Since the guidelines cover only concrete arches, one vulnerability level for iron/metal structures was added as historical structures are generally rated higher according to the guidelines' approach.	Iron/metal	High
		Other	Medium-high
Cable-stayed	The guidelines do not specify what class should be used for "Ponte strallato" (cable-stayed bridges). Hence, following the guidelines' suggestion, a conservative vulnerability class was assumed, assigning the highest class to all bridges, except for steel bridges, which were assigned the second-highest class, in line with the guidelines' typical approach of setting one class lower for steel bridges.	Other	High
		Steel	Medium-high
Cantilever	The guidelines do not specify what class should be used for "Ponte a sbalzo" (cantilever bridges). Hence, following the guidelines' suggestion, a conservative vulnerability class was assumed, assigning the highest class to all bridges.	Any	High
Truss	The classification "Ponte a travata reticolare" (truss bridges) was assummed following the material-based vulnerability hierarchy established in the guidelines.	Other	Medium-high
		Steel	Medium
Suspension	The guidelines do not specify what class should be used for "Ponte sospeso" (suspension bridges). Hence, following the guidelines' suggestion, a conservative vulnerability class was assumed, assigning the highest class to all bridges.	Any	High

Monitoring Capabilities

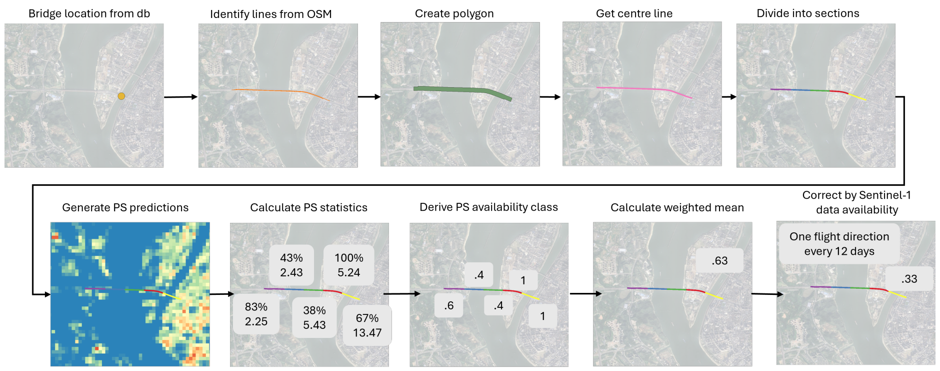

Supplementary Figure 4. Step-by-step process for assigning PS availability and spaceborne monitoring classes.

Discussion

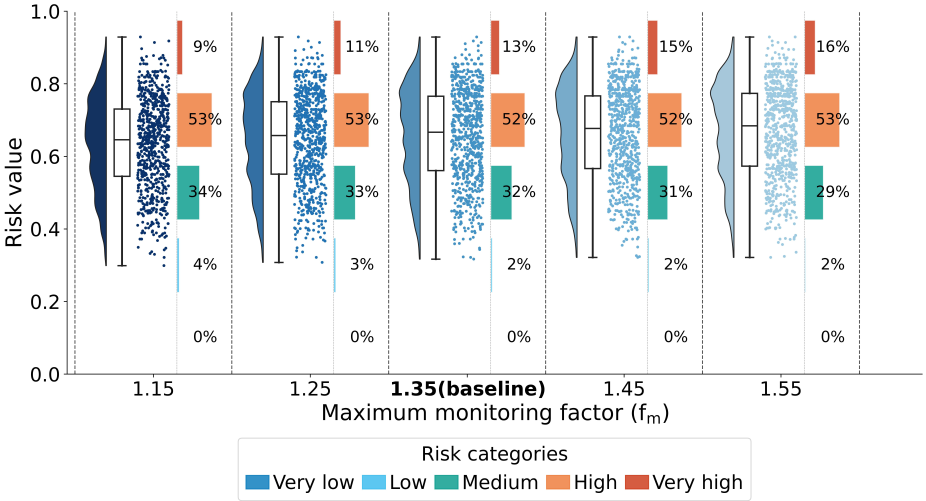

(a) Distribution of integrated geo-hazard risk values across different maximum monitoring factors (f_m).

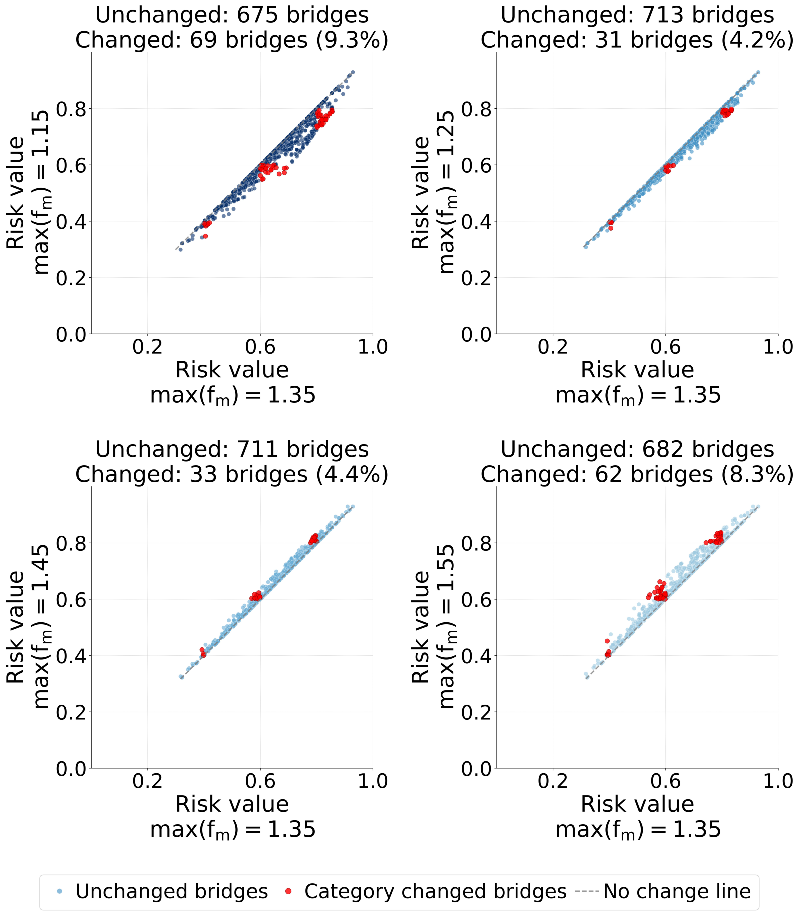

(b) Pairwise comparison of integrated risk values: baseline scenario ($f_m = 1.35$) versus alternative monitoring factors.

Supplementary Figure 5. Sensitivity analysis of the impact of the maximum monitoring factor (f_m) on geo-hazard risk integrated with combined SHM-spaceborne monitoring. Note: f_m denotes $f_{\text{monitoring}}$ used elsewhere in this paper.

References

1. Caprani, C. C. & De Maria, J. Long-span bridges: Analysis of trends using a global database. *Struct. Infrastructure Eng.* **16**, 219–231 (2020). DOI: 10.1080/15732479.2019.1639773.

2. Herrera-García, G. *et al.* Mapping the global threat of land subsidence. *Science* **371**, 34–36 (2021). DOI: 10.1126/science.abb8549.

3. Redshaw, P. & Bottomley, J. The Global Landslide Hazard Map: Final Project Report. Tech. Rep., The World Bank (2020).
